# Evaluating the Impact of a Web-Based Risk Assessment System (CareSage) and Tailored Interventions on Health Care Utilization: Protocol for a Randomized Controlled Trial

**DOI:** 10.2196/10045

**Published:** 2018-05-09

**Authors:** Ramya Sita Palacholla, Nils C Fischer, Stephen Agboola, Mariana Nikolova-Simons, Sharon Odametey, Sara Bersche Golas, Jorn op den Buijs, Linda Schertzer, Joseph Kvedar, Kamal Jethwani

**Affiliations:** ^1^ Partners Connected Health, Partners Healthcare Massachusetts General Hospital Harvard Medical School Boston, MA United States; ^2^ Philips Healthcare Eindhoven Netherlands; ^3^ Philips Lifeline Framingham, MA United States

**Keywords:** decision support techniques, algorithm, multiple chronic diseases, risk assessment, health services for the aged, emergency responders, hospitalization, patient readmission

## Abstract

**Background:**

Soaring health care costs and a rapidly aging population, with multiple comorbidities, necessitates the development of innovative strategies to deliver high-quality, value-based care.

**Objective:**

The goal of this study is to evaluate the impact of a risk assessment system (CareSage) and targeted interventions on health care utilization.

**Methods:**

This is a two-arm randomized controlled trial recruiting 370 participants from a pool of high-risk patients receiving care at a home health agency. CareSage is a risk assessment system that utilizes both real-time data collected via a Personal Emergency Response Service and historical patient data collected from the electronic medical records. All patients will first be observed for 3 months (observation period) to allow the CareSage algorithm to calibrate based on patient data. During the next 6 months (intervention period), CareSage will use a predictive algorithm to classify patients in the intervention group as “high” or “low” risk for emergency transport every 30 days. All patients flagged as “high risk” by CareSage will receive nurse triage calls to assess their needs and personalized interventions including patient education, home visits, and tele-monitoring. The primary outcome is the number of 180-day emergency department visits. Secondary outcomes include the number of 90-day emergency department visits, total medical expenses, 180-day mortality rates, time to first readmission, total number of readmissions and avoidable readmissions, 30-, 90-, and 180-day readmission rates, as well as cost of intervention per patient. The two study groups will be compared using the Student *t* test (two-tailed) for normally distributed and Mann Whitney U test for skewed continuous variables, respectively. The chi-square test will be used for categorical variables. Time to event (readmission) and 180-day mortality between the two study groups will be compared by using the Kaplan-Meier survival plots and the log-rank test. Cox proportional hazard regression will be used to compute hazard ratio and compare outcomes between the two groups.

**Results:**

We are actively enrolling participants and the study is expected to be completed by end of 2018; results are expected to be published in early 2019.

**Conclusions:**

Innovative solutions for identifying high-risk patients and personalizing interventions based on individual risk and needs may help facilitate the delivery of value-based care, improve long-term patient health outcomes and decrease health care costs.

**Trial Registration:**

ClinicalTrials.gov NCT03126565; https://clinicaltrials.gov/ct2/show/NCT03126565 (Archived by WebCite at http://www.webcitation.org/6ymDuAwQA).

## Introduction

Globally, the population of individuals 65 years and older is increasing rapidly. Older patients have higher health care expenditures with costs usually rising after the age of 65 before peaking in the early to mid-nineties [1]. This trend of increasing health care costs has led the United States (US) Congressional Budget Office to project that net Medicare spending will increase from 3.5% of the gross domestic product (GDP) in 2014 to 5.7% of the GDP in 2039 (US $595 billion and approximately US $1.1 trillion, respectively) [2]. This has important policy implications for Medicare [3]. Recent estimates show that older patients with multiple chronic diseases contribute to the vast majority of total Medicare expenditures [4-6], most of which is due to emergency and post-acute care for chronic conditions [6]. According to the Centers for Medicare and Medicaid Services (CMS), nearly a quarter of all admissions were considered avoidable [7]. Another recent analysis which evaluated longitudinal health care utilization in older patients over a 5-year period showed that 21% (1104/5258) of all admissions in their cohort were potentially avoidable [8].

Health care utilization is unevenly distributed among Medicare beneficiaries. A study analyzing trends in Medicare spending concentration over a 30-year period showed that as Medicare spending increased from US $14.5 billion to US $295.2 billion from 1975 to 2004, the top 5% and 1% of beneficiaries accounted for 43% and 15.5% respectively of all Medicare expenditure in 2004 (US $125 billion and US $45.3 billion, respectively) [9]. Using the 2011-2012 Medicare claims data, another study by Joynt et al segmented beneficiaries into 6 potentially actionable groups [10]. This study found that the frail elderly (age ≥65 years and the presence of at least two conditions indicative of frailty) were most likely to be in the highest cost segment with their costs driven by inpatient and post-acute care services. More recently, another study divided older patients based on their health care costs (from most to least expensive) into 3 segments and organized them into a cost pyramid comprising of the following categories: top 5%, middle (6%-50%) and bottom segments (51%-100%) [11,12]. The authors examined cost data from 2010 through 2015, studying a cohort of patients going through the health care system and evaluated their health care utilization from one year to the next [12]. The study demonstrated that the middle segment represented the most expensive segment over time [12].

Currently, many health care organizations employ intensive population health management strategies which are targeted at patients in the top segment of the cost acuity pyramid to control costs. One such example is the integrated care management programs (iCMP) at Partners Healthcare that provides specialized care to high risk patients with multiple comorbid conditions [13]. Due to the finding that these high-cost patients transition to lower cost segments in subsequent years, a modified strategy is to target population health interventions at patients in the middle segment. Therefore, in this study, we evaluate the impact of CareSage, a risk assessment system, combined with personalized and timely interventions on the health care utilization and associated costs in older patients in the middle segment of the health care cost pyramid.

The primary aim of this study is to assess the impact of the CareSage risk assessment system combined with tailored interventions on the 180-day emergency department visits in a cohort of patients receiving care at a home health agency. Secondary aims include evaluating the impact of the CareSage risk assessment system and tailored interventions on the following: (1) 90-day emergency department visits, (2) 30-, 90-, and 180-day readmissions, (3) the total number of readmissions and total number of avoidable admissions, (4) total medical expenses and total expenses attributable to avoidable admissions, (5) mortality rates, and (6) time to first readmission.

## Methods

### Study Design

**Figure 1:**
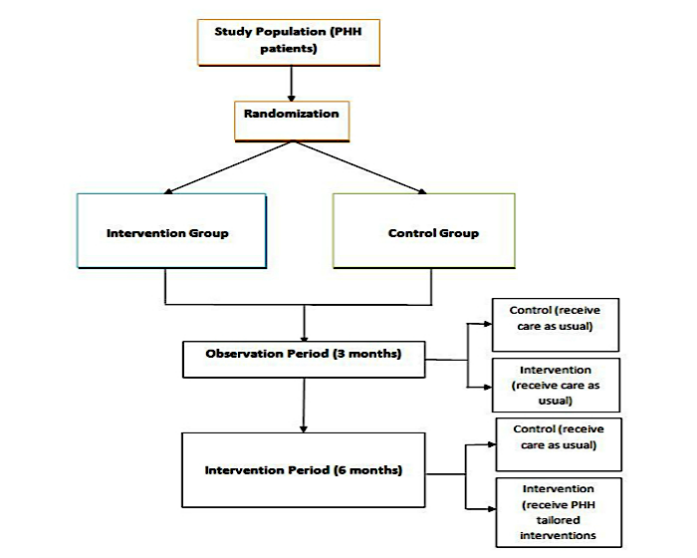
Research design, including a 3-month observation period followed by 6 months of the intervention. PHH: Partners Healthcare at Home.

The study will be implemented as a two-arm randomized controlled trial with two study periods, namely an initial 3-month observation period followed by a 6-month intervention period. Each participant will be in the study for a total of 9 months. At the beginning of the observation period (baseline), enrolled patients will be randomized into two groups, either the intervention or control group. During the observation period, the CareSage algorithm will calibrate using patient data (from both groups) collected from a Personal Emergency Response Service (PERS) device. During the 3-month observational period, all participants will complete the baseline study assessments and will continue to receive usual care from their care providers, but no study interventions will be administered. The 6-month intervention period begins immediately after the observational period. During the intervention period, patients in the intervention group will be actively monitored by CareSage and will receive personalized interventions when flagged as “high” risk for emergency transport. Patients in the control group will receive care as usual during the intervention period. Assessments of study outcomes will be made at the end of the 9-month study period. Figure 1 shows the research design.

### Participants

A total of 370 participants will be recruited from a pool of patients receiving care at Partners Health at Home (PHH)—a home health service that offers general care as well as specialized services to help patients manage chronic conditions. PHH consists of a network of qualified clinicians who deliver care designed to meet the unique needs of each patient, with the goal of optimizing independence and quality of life. On average, most patients are on PHH service for 2-3 months, but the duration may vary based on the patients’ condition and needs. Patients who are considered likely to benefit from PHH services are typically referred to the program by their care providers. PHH services patients who are receiving care from any of the Partners Healthcare Network of hospitals (comprising of 7 major hospitals, including 2 large academic centers and several community health centers) are eligible for this study. In addition, a subgroup of patients from PHH who may be enrolled in other care management programs such as iCMP will be included in the study.

During the study, staff will periodically review previous PHH admissions and identify potential study participants. Identified patients will be prescreened over the phone to gauge interest and confirm eligibility. Eligible and interested patients will be enrolled by study staff at the patient’s home. Screening, informed consent, randomization and survey completion will occur at this enrollment visit. During this visit, patients will be given a PERS device to use for 9 months. Patients will be instructed to wear the device continuously throughout the study period.

### Ethics Approval and Consent to Participate

Study was approved by the Partners Human Research Committee, the Institutional Review Board of Partners HealthCare, on 03/15/2017.

### Participant Inclusion and Exclusion Criteria

Eligible participants are aged 65 years or older, English speaking patients who are receiving care or recently (within 3 months) discharged from PHH, and whose total health care costs fall within the middle 6th to 50th percentile (the middle segment of the cost acuity pyramid) of all patients seen at Partners Healthcare during the fiscal year 2016. The middle segment cutoffs for 2016 were projected based on 2011-2015 data from the analysis that was the precursor to this prospective study. Patients will not be excluded based on their disease condition as long as they fulfill the inclusion criteria stated above. Patients with artificial pacemakers and other implanted devices (as a precautionary measure against possible interference with the PERS device), currently on admission in a hospital or a long-term-care facility including Skilled Nursing Facilities, having severe dementia, Alzheimer’s disease or any other serious psychiatric illness (severe anxiety disorder or psychosis) will be excluded from this study.

### Intervention

All study participants, irrespective of study group (control or intervention), will receive the following:

- A PERS device: This is a personal health technology designed to promote independent living in older adults by providing help in case of medical emergencies that lead to costly emergency department visits and hospitalizations. The PERS consists of 3 components: (1) a help push button that is worn as a necklace for 24 hours a day, (2) an in-home communication system, and (3) an emergency response center. Participants may press the help button at any time to activate the in-home communication system that connects to the response center. The response center associate enquires about the situation and contacts either an informal responder (eg, neighbor, a family member) or an Emergency Medical Service (eg, ambulance, police, or fire department) based on patient’s specific situation, and then follows up to confirm that help has arrived. All participants are instructed to directly contact the Lifeline call center by pushing the PERS button or call 911 if they experience worsening of symptoms or require immediate attention. The response center associate documents the incident data and registers the situation (eg, fall, respiratory problems, chest pain, social call) and the outcome (subscriber status, responder assistance, emergency hospitalization) using proprietary software. Participants are expected to use the PERS as needed during the study period.
- CareSage: In a previous study, the medical alert pattern data captured by the PERS service was used to develop and validate a risk prediction algorithm based on the user’s interaction with the device [14]. This algorithm is used by CareSage (a Web-based platform) to conduct risk assessments on patients was originally developed after studying a large cohort of the PERS subscribers (approximately 600,000) [14]. Thereafter, the algorithm was validated among a cohort (N=3335) of PHH patients to predict emergency transports in this population (area under the curve=.76). This algorithm was further refined with electronic medical records data and integrated into the health care provider-facing system (CareSage) that assesses a patient’s 30-day risk for emergency transport [11]. CareSage can assess a patient’s level of risk for emergency transport thereby providing an opportunity to intervene before an adverse event (emergency department visit, hospitalization) occurs. A snapshot of the provider facing Web portal is provided in Multimedia Appendix 1. CareSage is an analytics engine that builds on real-time and historical data about patients collected from both providers and Lifeline services. 

Participants in the control group will receive the following:

- Passive Monitoring: These participants will continue to use the PERS system. After the 3-month observation period, the CareSage algorithm will continue to only passively monitor control group participants but will not alert the study nurse about their risk for emergency transport. The data from CareSage will continue to be captured at the backend and will be collected at the end of the study.
- Care as usual: Participants will continue to receive medical treatment and discuss any questions or concerns with their care providers as usual. There will be no triage for control group participants.

**Figure 2:**
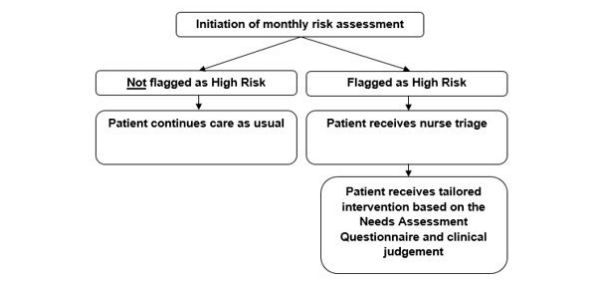
Stepped care approach for those flagged as high risk and not flagged as high risk.

Participants in the intervention group will receive the following:

- Active monitoring: Patients in the intervention group will be assessed by the Web-based CareSage risk assessment system, which can identify when a patient is at risk for emergency transport in any upcoming 30-day period. Patients identified as “high risk” will receive nurse triage calls and, depending on their needs, tailored interventions per a stepped-care approach (Figure 2). The principal goals of the intervention will be to address knowledge gaps in disease self-management, ensure compliance with medications and healthy diet, and identify recurrent symptoms amenable to treatment on an outpatient basis to prevent readmissions.
- Nurse Triage Call*:* During the intervention period, “high-risk” patients will be called by a trained study nurse to assess the physical and psychological patient’s needs using a health needs assessment guide (Multimedia Appendix 2) developed by the study team. These 15-30-minute telephone calls will focus on how the patient is feeling, adherence to treatment plans, and connect the patient to any available resources (primary care physician, dietician etc) as needed.

**Figure 3:**
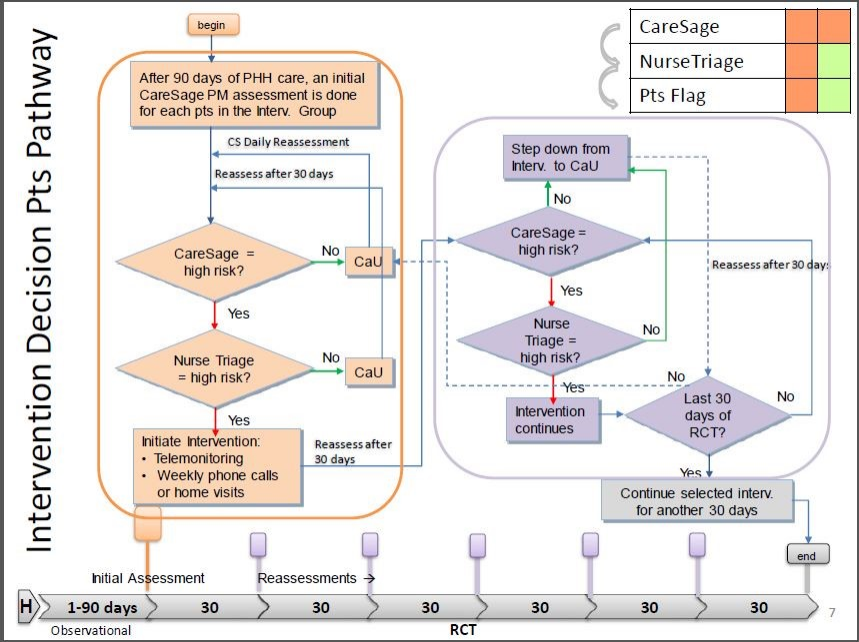
Details of the intervention decision pathway which includes patient education sessions, home visits, and telemonitoring support for patients classified as high risk in each 30-day assessment during the intervention period. CaU: care as usual; CS: CareSage; Pts Flag: Patients Flagged; PHH: Partners Healthcare at Home; RCT: randomized controlled trial.

Tailored PHH Interventions The PHH interventions consist of multiple components tailored to individual patients based on the needs identified during nurse triage call. The complete intervention decision pathway is depicted in Figure 3. There are 3 interventions that the study nurse may offer.

The first intervention is patient education where participants receive structured weekly telephone-based education sessions over the next 4-week period. In addition to the structured educational sessions, patients may receive a “just-in-time” teaching session based on the clinical judgment of the study nurse. Patient education will cover a variety of topics including diet, physical activity, importance of daily measurements, recognizing symptoms of disease, and medication adherence. The study nurse will also assess current medications and make specific recommendations to eliminate unnecessary medications and simplify the overall regimen. Structured education sessions will typically last for 30-45 minutes over a telephone call.

The second intervention is home visits. If the patient needs further intervention other than education, the study nurse may contact the patient’s primary care provider (PCP) to get approval for an in-person clinical assessment. If the PCP’s approval is received, then the bulk of the patient’s education covering pertinent topics such as diet, activity, medications, and other topics would occur during or after the in-person clinical assessment.

If the study nurse does not receive an approval for the in-person clinical assessment, the patient still receives basic education intervention from the nurse (as outlined above). If the PCP orders the patient to be sent to the emergency department or feels the patient needs immediate attention, the same advice will be communicated to the patient by the study nurse. In all scenarios, the PCP’s decision supersedes any other study assessment.

The third and final intervention is telemonitoring support. During the phone call assessment (nurse triage), the study nurse may determine the need for telemonitoring support for a patient. Before providing telemonitoring support, the nurse will contact the patient’s PCP to obtain approval. Within 3-4 business days starting from the day of PCP approval, study staff will install the telemonitoring equipment at the participants’ residence with explanations on how to use the technology. The support will be maintained for a period of 4 weeks until the patient is reassessed by the CareSage algorithm. During the period of telemonitoring support, patients monitor relevant physiologic parameters (blood pressure, heart rate, weight, and blood oxygen saturation) and answer questions on heart failure-related symptoms on a touch-screen computer daily. The remote monitoring equipment includes easy-to-use devices approved by the Food and Drug Administration, namely a monitor, a digital weight scale, a blood pressure cuff and meter, and a pulse oximeter device. If the patient’s PCP does not approve telemonitoring support, the patient still receives basic patient education (described above) from the study nurse. Data from these devices will be transferred securely to a remote monitoring database where the records are reviewed by the study nurse daily.

All study participants will be instructed to contact the Lifeline call center by pushing the PERS button device or call 911 directly if there is worsening of symptoms during the study period that require immediate attention.

### Outcomes Measurement

The number of 180-day (6-month intervention period) emergency department visits in the intervention and control group will be measured as the primary outcome of interest for this study. Secondary outcomes will include the number of 90-day emergency department visits, the total cost of intervention provided to patients, total medical expenditure incurred during the study period (including total expenses attributable to the avoidable admissions), 180-day mortality rate, and time to first readmission. Furthermore, health care usage including emergency transport use, readmissions (30-, 90-, and 180-day), and the total number of avoidable admissions will be evaluated at the end of the study period. Following the methodology cited in Walsh et al and Segal et al, potentially avoidable admissions will be determined by analyzing the principal diagnosis ICD-9 code associated with each inpatient admission and categorizing the codes into 3 groups which are (1) those potentially avoidable in both institutional and non-institutional settings, (2) those potentially avoidable only in institutional settings, and (3) those which are not determined to be potentially avoidable [7,15].

### Data Collection

All study data will be aggregated and analyzed at the end of the study period. Data will be collected at enrollment and closeout as described in  Multimedia Appendix 3. Patient demographics will be collected using an enrollment questionnaire developed by the study investigators. All data pertaining to health care utilization (emergency department visits, readmissions during study period, avoidable admissions, mortality, and total medical expenditures) will be extracted from the Partners Healthcare patient databases—Enterprise Data Warehouse and the Remote Patient Data Registry. The total cost of intervention provided to patients by PHH will be sourced from the PHH program costs. The emergency transport use data will be collected from the Phillips Lifeline database.

The Remote Patient Data Registry is a centralized clinical data warehouse that operates under Partners Research Information Services. It securely stores all data from across Partners hospital systems in one place and ensures security and confidentiality of patient information [16]. The Enterprise Data Warehouse is a single repository of clinical, operational, financial, and claims data of patients receiving care across the Partners system. The Phillips Lifeline database contains historical data such as demographics, patients’ living situations and care giver network, self-reported medical conditions, and emergency transport data from the PERS device. The care-giver network information includes number of responders, the number of people who live with the patients, and persons to be notified after an emergency transport. Emergency transport data from the PERS device includes all information gathered during the interactions of the patients with the Lifeline call center associates. For all calls, the situation (eg, fall, respiratory problems, chest pain, and social call) and the outcome (eg, subscriber OK, responder assistance, and emergency hospital transport) are collected by the associate via custom-made software. All other data collected via questionnaires is stored in a secure electronic database, REDCap, which is a secure Web application for building and managing Web-based surveys and databases.

Patients meeting the initial eligibility criteria are identified through an Enterprise Data Warehouse query. The query will be written in structured query language to identify patients who meet the pre-set criteria. PHH study staff acquire a daily list of medical record numbers of newly-admitted patients, and then run the query for that distinct list of patients. The query output summarizes the patients’ age, 2016 health care costs, and determines whether their health care costs place them in the middle cost acuity segment. Eligible patients will be contacted by study stuff to be enrolled into the study if interested.

### Analysis

#### Sample Size

Based on a similar study with a comparable population by Coleman et al, which reported an odds ratio of 0.57 for 180-day hospital visits compared to matched controls, we are expecting to reduce the number of emergency department visits within a 180-day period by at least 35% in the intervention group compared to controls [17]. At 80% power and a type I error (alpha) of .05, we will need 185 participants in each of the study arms, accounting for a lost to follow-up of 15%.

#### Randomization

A total of 370 participants will be enrolled and randomized (via a computer program) to one of two groups (intervention group or control group) in a ratio of 1:1 (185 participants per group). Randomization will utilize random permutated blocks to optimize balance in each treatment group at any point in time during the study. Treatment allocation will be concealed in an opaque envelop that will be opened after the informed consent procedures. Neither the patient nor the members of the study team will be aware of the treatment assignment until after randomization.

#### Statistical Analysis Plan

Data collected will be analyzed with data analysis (STATA, version 14) and statistical software (R, version 3.4.1). All the analyses will be conducted per the intention-to-treat principle. Descriptive statistics will be used to characterize the study sample, and we will summarize participants’ characteristics by study arm. The two study groups (intervention and control) will be compared by the Student *t*-test (two-tailed) for normally distributed and the Mann Whitney *U* test for skewed continuous variables, respectively. The chi-square test will be used for categorical variables. Time to event (readmission) and 180-day mortality between the two study groups will be compared by using the Kaplan-Meier survival plots and the log-rank test. Cox proportional hazard regression will be used to compute hazard ratio and compare outcomes between the two groups. We will also conduct subgroup analyses based on enrollment in iCMP during the 6-month intervention period. Fixed and per-patient intervention costs will be calculated from PHH records. The monthly per-patient costs of health care services and out-of-pocket costs will be combined, and multivariate analyses comparing this sum (potential cost offset) for intervention versus usual care patients will be conducted.

## Results

### Enrollment and Study Completion

Active enrollment of participants for this trial is currently underway and the study is expected to be completed by end of 2018. The study results are expected to be published in early 2019.

### Availability of Data and Material

The data that support the findings of this study are available from Partners Healthcare but restrictions apply to the availability of these data, which were used under license for the current study, and so are not publicly available. Data are however available from the authors upon reasonable request and with permission of Partners Healthcare Institutional Review Board.

## Discussion

### Study Rationale

The goal of this study is to determine whether a risk assessment system (CareSage) combined with targeted interventions for high-risk patients can potentially decrease health care utilization and the associated costs. Population health management interventions have traditionally focused on the most expensive patients [18,19] and have not taken into consideration the dynamicity of the various costs segments as demonstrated by Niklova-Simons et al [12]. This study, however, is uniquely geared towards preventing patients in the middle segment of the cost acuity pyramid from transitioning to the higher cost segment. Furthermore, the current landscape of a rapidly aging population with multiple comorbidities presents numerous challenges to effective care management in this group. Therefore, as developed countries’ populations age and the number of associated chronic health conditions increase, alternatives to hospital and institutional care are needed to optimize health care costs and improve patient outcomes [20]. 

Two key strategies are evaluated in this study. First, patients at high risk for emergency transport are identified using the CareSage risk assessment system, which leverages predictive analytics using remotely collected data from patients at home. Rather than targeting every patient currently in a home-based care management program or discharged from a care management program, the CareSage can identify those patients that need the most help managing their conditions. Recent studies have reported the development and validation of predictive models that can be used to systematically identify individuals at high risk for an unfavorable outcome in a hospital setting [21-23]. Such risk assessment models have great potential to inform treatment decisions and improve the quality of care delivered to patients who are receiving care at home. Thus, the CareSage risk assessment system presents a unique opportunity to efficiently allocate limited health care resources to patients that need them the most and thereby reduce costs associated with excessive use.

The second strategy is delivering targeted interventions (per established protocols) to patients identified as “high risk” by the CareSage predictive algorithm. Interventions will be adapted to fit the patient’s needs with a focus on the patient’s general functioning, including physical and psychological health status. Several studies have evaluated the effect of personalized multidisciplinary interventions for chronic conditions including heart failure, depression, and risk of falling in seniors [24,25]. Results showed significant reductions in health care utilization as well as improved patient quality of life. Therefore, we hope that an integrated approach to patient care management will have a significant impact on overall health care utilization and costs in older patients with multiple chronic diseases.

This study has a few limitations. First, the target population consists of English-speaking elderly patients who are receiving care from a home health care agency, thereby limiting the generalizability of the study results to other populations. The applicability of our study findings to other settings will require further investigation but, nevertheless, the findings will set the stage for future studies that could potentially target a more diverse population. Second, it might be difficult to capture data on hospitalizations that may occur outside of the Partners Healthcare network of hospitals. Capturing emergency department visits and hospitalizations outside of the Partners network is important because the study population is a high health care utilization group which is at risk for emergency transport and patients are typically taken to the nearest medical center for emergency care services. However, the Phillips Lifeline call center data and self-reported data from patients may help mitigate some of these challenges.

### Conclusions

The CareSage risk assessment system provides an opportunity to identify and target interventions at patients at high risk for emergency health care utilization. The efficacy of this novel patient care approach, if proven, may present a feasible and relatively inexpensive means to improve overall patient outcomes and decrease costly health care utilizations.

## Appendix

Multimedia Appendix 1 Screen shot of provider-facing portal which shows risk categories for each participant.

Multimedia Appendix 2 Needs assessment questionnaire.

Multimedia Appendix 3 Data collection schedule.

## Abbreviations

CaU: care as usual
CS: CareSage
CMS: Centers for Medicaid and Medicare Services
EMR: electronic medical record
Pts Flag: Patients Flagged
GDP: gross domestic product
iCMP: Integrated Care Management Program
PCP: primary care provider
PERS: Personal Emergency Response Service
PHH: Partners Healthcare at Home
