# Supplementary material for: Evaluating the Impact of a Web-Based Risk Assessment System (CareSage) and Tailored Interventions on Health Care Utilization: Protocol for a Randomized Controlled Trial
Source: JMIR Res Protoc. 2018 May 9;7(5):e10045. doi: 10.2196/10045 (PMC5966651; doi:10.2196/10045)
Supplement: Multimedia Appendix 1 [file resprot_v7i5e10045_app1.pdf]

Appendix 1

Part A: Screen shot of provider-facing portal which shows risk categories for each participant.

PHILIPS
Lifeline

Risk for Transport Report

As of: 9/22/2015

Patients with risk for transport - NH123

High Risk (6.00-5.60) | Medium Risk (5.59-3.40) | Low Risk (3.39-0.60) | Slight Risk (0.59-0.00)

First Name	Last Name	MRN	DOB	Phone Number	Days On Service	Risk Transport Score	Risk	Alerter
**Able HomeCare - NH100**								
Flossie	Alans	BGH12345678906	02/15/1901	(xxx) xxx-7049	312	0.19	Slight	
Josie	Smith	BGH12345678900	04/04/1902	(xxx) xxx-8187	56			< 60 days on service
Morton	Thompson	BGH12345678901	03/16/1903	(xxx) xxx-5121	221	0.08	Slight	
**Clay HomeCare - NH101**								
Florine	Wiggins	as_enr_test2	05/18/1904	(xxx) xxx-7152	75	0.10	Slight	
Nikki	Wiggins	as_enr_test3	09/28/1905	(xxx) xxx-8663	91	0.09	Slight	
Maritza	Wiggins	as_enr_test1	03/11/1906	(xxx) xxx-8668	151	0.09	Slight	
Nikki	Wiggins	as_enr_test3	09/28/1907	(xxx) xxx-8663	146	0.08	Slight	
**Northern HomeCare - NH102**								
Orville	Mackie	BGH12345678903	01/31/1920	(xxx) xxx-4636	122	0.08	Slight	
**VIP HomeCare - NH103**								
Miranda	Norton	BGH12345678904	03/01/1921	(xxx) xxx-6058	82	6.00	High	New High Risk
**York HomeCare - NH104**								
Marcelino	Cappella	BGH12345678905	12/22/1922	(xxx) xxx-1858	114	0.08	Slight	

)ata is based upon the risk ranges and algorithm sensitivity as set by the Health Care Organization. Lifeline Systems Company DBA Philips Lifeline makes no claims tha any person not specifically identified as a risk for transport should not be transported.

Page 1 of 1
