# Supplementary material for: Evaluating the Impact of a Web-Based Risk Assessment System (CareSage) and Tailored Interventions on Health Care Utilization: Protocol for a Randomized Controlled Trial
Source: JMIR Res Protoc. 2018 May 9;7(5):e10045. doi: 10.2196/10045 (PMC5966651; doi:10.2196/10045)
Supplement: Multimedia Appendix 2 [file resprot_v7i5e10045_app2.pdf]

Appendix 2

Needs Assessment Questionnaire

"Hi, this is nurse X. I am from the Phillips Lifeline CareSage study team. Would you have a few minutes to do a quick check-in on your health as a part of the study?

If patient response is:
a. "No"- Schedule another triage call at a time convenient for the patient.
b. "Yes"- Continue assessment using the questions below

Section A: Patient Information (pre-populated)

1. What is your name?

____

2. Who is your Primary Care Provider Name?

____

3. Date of Assessment:

____

Section B: General Health Information

1. How are you feeling today?

____
____
____

2. Have you seen your PCP lately? Why?

____
____
____

3. If **NO**, do you have any visits to the PCP scheduled?

____
____

4. Are you currently experiencing any health problems?

____
____
____

5. Are you taking all the medications prescribed by your doctor?

6. Do you need a refill of any of your current medications?

Notes:

**Reviewed list of current medications:**<br>• Yes<br>• No	**Status:**<br>• Change<br>• No change	**Clinical Recommendations:**

Section C: Risk Assessment

General Symptom Assessment

1. Have you had any fever in the last 72 hours?

2. Have you noticed any changes in your appetite (overeating or not eating enough)?

3. Do you currently experience nausea or vomiting after eating?

-

4. Do you feel any fatigue/ tiredness lately?

-

5. Have you experienced any dizziness or black outs lately?
____-
____

Activity Assessment

1. Do you engage in exercise at least two to three times in a week?

____

____

2. Describe the type of exercise?

____
____

If the patient does not exercise, then:

3. Do you understand how regular exercise can benefit you?
____-
____

4. Have any of your physicians spoken to you about the benefits of exercising regularly?
____
_-
____

Respiratory Symptom Assessment

1. Are you currently experiencing any shortness of breath/ difficulty in breathing?
____
_-
____

2. Do you have currently have any cough?
____
_-
____

3. Can you climb up a flight of stairs or walk at least 100 yards without losing your breath?

____-

____

4. Do you feel any tightness in your chest?

____-

____

5. Do you feel tired all the time or for the most part of the day?

____-

____

Mental Health Assessment (PHQ-2)

1. Over the past 2 weeks, have you been bothered by having little or no pleasure in doing things? (**Score 0-3**, 0= Not at all, 1= several days, 2= More than half days, 3= Nearly every day)

____-

____

2. Over the past 2 weeks, have you been feeling down, depressed, hopeless? (**Score 0-3**, 0= Not at all, 1= several days, 2= More than half days, 3= Nearly every day)

____-

____

Daily Activity Assessment

1. Are you currently experiencing any difficulty doing your activities of daily life such as dressing, eating, bathing?

____-

____

2. Do you have troubling falling asleep?

____
_-
____

Pain Assessment

1. Are you currently experiencing pain in any part of your body (except minor headaches, toothaches and sprains)?

____
____

If patient complains of pain:

2. Is this a new or recurring pain?

____
_-
____

3. Where is the site of the pain?

____
____

3. Describe the pain: Is it sharp, dull, aching, burning, or crushing?

____
____

4. On a scale of 1 (mild) to 10 (worst pain you can imagine), please rate the intensity of your pain.

____
____

5. Are you currently receiving any medication for your pain? Who prescribed it?

____
_-
____

Bladder Control Assessment

1. Do you have a sudden urge to urinate with no or little warning?

____

____

2. Do you experience any pain while urinating?

____

____

3. Do you feel that you urinate very frequently during the day?

____

____

Final Recommendations and Management Plan

Please choose a follow up action for the patient.

a. No further action required
b. Education session (on telephone) required
c. Further assessment required by PCP
d. TM support required (for CHF patients)
e. Deteriorating disease symptoms- contact PCP immediately
f. Other, specify

____

Comments/ next steps:

____

____

____

____
