# Supplementary material for: Evaluating the Impact of a Web-Based Risk Assessment System (CareSage) and Tailored Interventions on Health Care Utilization: Protocol for a Randomized Controlled Trial
Source: JMIR Res Protoc. 2018 May 9;7(5):e10045. doi: 10.2196/10045 (PMC5966651; doi:10.2196/10045)
Supplement: Multimedia Appendix 3 [file resprot_v7i5e10045_app3.pdf]

Table 1 Data Collection Schedule

	Variables	Enrollment	Close-out	Data Source
1.	Demographics, PHQ-8	X		Enrollment Questionnaire
2.	90, 180 day ED visits		X	RPDR/ EDW
3.	30, 90, 180 day readmissions		X	RPDR/ EDW
4.	Emergency Transport use		X	PLL-database
5.	Total avoidable admissions		X	RPDR/ EDW
6.	TME and total expenses attributable to avoidable admissions		X	RPDR/ EDW
7.	Total and per-patient cost of intervention		X	PHH program costs
8.	180-day mortality rate		X	RPDR/ EDW
9.	Time to first readmission		X	RPDR/ EDW
